# Differential subordination and superordination results for *p*-valent analytic functions associated with (*r,k*)-Srivastava fractional integral calculus^✰^

**DOI:** 10.1016/j.mex.2024.103079

**Published:** 2024-11-30

**Authors:** Adel Salim Tayyah, Waggas Galib Atshan

**Affiliations:** Department of Mathematics, College of Science, University of Al-Qadisiyah, Diwaniyah 58002, Iraq

**Keywords:** Analytic function, P-valent function, Fractional calculus, Srivastava fractional integral operator, (r,k)-gamma function, Hypergeometric function, Subordination, Superordination, Hadamard product, Vortex motion, Differential Subordination and Superordination involving (r,k)-Srivastava Fractional Integral Calculus

## Abstract

The object of the present paper is to investigate generalizations of the hypergeometric function and Srivastava fractional integral calculus by using a general version of gamma function(namely (r,k)-gamma function).

• Some fundamental results for these new concepts are provided.
• We introduced differential subordination and superordination results associated with the defined new fractional integral operator.
• Also, we establish sandwich results for p-valent analytic functions involving this operator.
• Finally, an application to fluid mechanics is discussed.

## Graphical abstract

**Figure:**
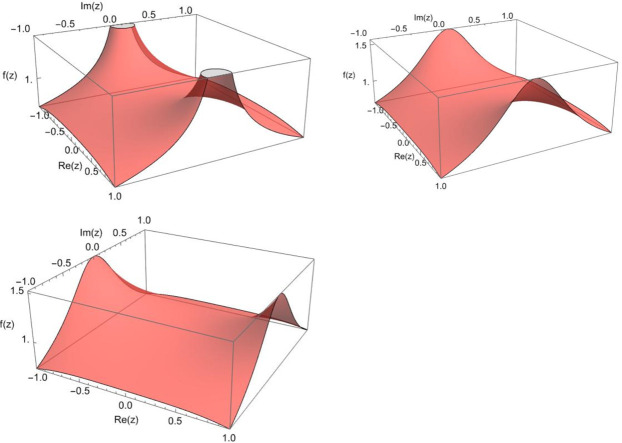

Specifications table

*This table provides general information on your method.*

**Table:** 

Subject area:	Mathematics and Statistics
More specific subject area:	Fractional Calculus and Analytic Functions
Name of your method:	**Differential Subordination and Superordination involving**(r,k)**-Srivastava Fractional Integral Calculus**
Name and reference of original method:	**Differential Subordination:** **S. S. Miller and P. T. Mocanu, Second order differential inequalities in the complex plane, J. Math. Anal. Appl. 65(1978), 289–305.**
Resource availability:	*Not applicable).*

## Background

This paper aims to generalize Srivastava fractional integral calculus through the (r,k)-hypergeometric function. By establishing fundamental results related to this function, we enhance the theoretical framework of fractional calculus.

The motivation for providing this methodology stems from the need to advance the existing understanding of fractional calculus and its applications. We introduce a new differential subordination and superordination results associated with a newly defined fractional integral operator can be used in many areas, such as fluid mechanics, by showing new results for fractional subordination . Additionally, we present sandwich results for p-valent analytic functions, highlighting the utility of the new operator in formulating relevant theorems.

By employing the (r,k)-gamma function, we reformulate the fractional integral operator, broadening the scope of fractional calculus and its applications in complex analysis. This research lays the groundwork for future studies on the (r,k)-hypergeometric function, contributing to advancements in mathematical analysis within fractional calculus and related areas.

## Method details

### Introduction and preliminaries

Analytic function theory is an ancient subject that originated around the beginning of the century. Moreover, in recent years, it has undergone swift and ongoing advancements. Among its prominent topics is the study of the geometric and analytic properties of certain common types of analytic functions, known as univalent and multivalent functions within unit disk U={z∈C:|z|<1}. One of the important topics of analytic function theory is the establishment of differential subordination and superordination, which examine how one analytic function affects another.

The journey of development in differential subordination began in 1974 with the study of properties of univalent functions by Miller et al. [1], where he established

$$Re\left(f\left(z\right)+\alpha \frac{zf^{\prime }\left(z\right)}{f\left(z\right)}\right)>0,\;\text{implies}\;Re\left(f\left(z\right)\right)>0.$$

Subsequent studies have yielded new insights in analytic function theory, with ongoing active research in this field as evidenced by recent literature [[2], [3], [4], [5], [6], [7], [8], [9], [10], [11], [12]]. Essentially, the concepts of subordination and superordination are very important, serving as basic tools for complex field C. Given the challenges of using traditional comparisons ≥ (or ≤) in this context, these concepts provide an alternative that enables functions to adapt to imposed constraints. Subordination bounds one function by another, allowing us to describe its behavior and properties, as well as calculate its Taylor-Maclaurin coefficients. Additionally, these concepts give Sandwich theorem, which provides a framework for bounding the properties and behavior of a function within range defined by two other functions.

On the other hand, fractional calculus studies generalizations of the basic operators of ordinary integration and differentiation, which is defined by allowing their order from integer domain to complex domain. Many researchers have proposed numerous generalizations for fractional differentiation and integration, such as [[13], [14], [15], [16], [17]].

After mentioning the theory of analytic functions, geometric functions, and differential subordination, as well as fractional calculus. We now highlight studies that have extended these topics by using the concepts of fractional differentiation and integration; for instance, see [6,[18], [19], [20], [21], [22], [23], [24], [25], [26], [27], [28]]. In our work, we generalized the hypergeometric function pFq (see [29]) and subsequently utilized it to generalize the Srivastava fractional integral calculus $I_{z}^{\lambda ,\delta ,\sigma }$ detailed in [23]. Therefore, we recall these definitions pFq and $I_{z}^{\lambda ,\delta ,\sigma }$ as follows: for $a_{j}\in C,\;j=1,2,\ldots ,p$ and $b_{k}\in C\setminus {Z_{0}}^{-},\;k=1,2,\ldots ,q$, the extended hypergeometric function is given by

(1.1) $$pFq\left(a_{1},\ldots ,a_{p};b_{1},\ldots ,b_{q};z\right)=\sum_{n=0}^{\infty }\frac{{\left(a_{1}\right)}_{n}\ldots {\left(a_{p}\right)}_{n}}{{\left(b_{1}\right)}_{n}\ldots {\left(b_{q}\right)}_{n}}\frac{z^{n}}{n!},\;\left(p\leq q+1;\;p,q\in N_{0}\right),$$

where ${\left(a\right)}_{n}$ is the Pochhammer symbol given by

$${\left(a\right)}_{n}=\frac{\Gamma \left(a+n\right)}{\Gamma \left(a\right)}=\left\{ \begin{array}{c} 1,\;n=0\\ a\left(a+1\right)\left(a+2\right)\ldots \left(a+n-1\right),\;n\in N \end{array}\right.\;.$$

For $\lambda \in R^{+}$, δ,σ∈R, the fractional integral operator $I_{z}^{\lambda ,\delta ,\sigma }$ is given by

(1.2) $$I_{z}^{\lambda ,\delta ,\sigma }\;f\left(z\right)=\frac{z^{-\lambda -\delta }}{\Gamma \left(\lambda \right)}\int_{0}^{z}{\left(z-\zeta \right)}^{\lambda -1}\times \;2F1\left(\lambda +\delta ,-\sigma ;\lambda ;1-\frac{\zeta }{z}\right)\;f\left(\zeta \right)d\zeta ,$$

where f(z) is an analytic function in a simple connected region of C containing 0 with order $f\left(z\right)=o\left({\left|z\right|}^{\epsilon }\right)$, z→0, and ϵ>max{δ−σ,0}−1.

Refer to [30] for all the bellow mentioned concepts. Let H(U) denote the class of analytic function in U and H[a,n] the subclass of H(U) represented by

(1.3) $$f\left(z\right)=a+\sum_{n=p}^{\infty }a_{n}z^{n}\;$$

The p-valent (normalized) analytic functions are defined $A_{p}=H\;\left[0,p\right]$(A=H[0,1]) with $a_{p}=1\left(a_{1}=1\right)$, respectively. Denote by S, the subclass of A which contains all univalent functions (analytic and one-to-one functions) in U.

For a function f is purported to be subordinate to g (g is superordinate to f) and denoted by f≺g if there is an analytic function w:U→U with w(0)=0 and |w(z)|<1 (z∈U) implies f(z)=g(w(z)). Equivalently, f(0)=g(0) and f(U)⊂g(U) (see [31,32]).

Let $\psi \left(r,s,t;z\right):C^{3}\times U\to C$ and h∈S. If ξ is analytic in U and satisfies

(1.4) $$\psi \left(\xi \left(z\right),\;z\xi^{\prime }\left(z\right),\;z^{2}\xi^{\prime \prime }\left(z\right);z\right)≺h\left(z\right),\;$$

hence ξ is referred to as a solution of (1.4). Let χ∈S with ξ≺χ for all ξ satisfying (1.4); then the function χ is called a dominant of the inequality. The best dominant is $\overset{⌣}{\chi }$ that it corresponds to $\overset{⌣}{\chi }≺\chi$ for all dominant χ of (1.4).

Let $\psi \left(r,s,t;z\right):C^{3}\times U\to C$ and h are analytic functions. If ξ and $\psi (\xi \left(z\right),\;z\xi^{\prime }\left(z\right),\;z^{2}\xi^{\prime \prime }\left(z\right);z)$ are belong to S and satisfy

(1.5) $$h\left(z\right)≺\psi \left(\xi \left(z\right),\;z\xi^{\prime }\left(z\right),\;z^{2}\xi^{\prime \prime }\left(z\right);z\right),\;$$

hence ξ is referred to as a solution of (1.5). Let χ an analytic function with χ≺ξ for all ξ satisfying (1.5); then the function χ is called a subordinate of (1.5). The best subordinate is $\overset{⌣}{\chi }$ that corresponds to $\chi ≺\overset{⌣}{\chi }$ for all subordinate χ of (1.5).

Following Miller and Mocanu [30,33], Bulboacă [34] investigated certain classes of first order differential subordinations as well as superordinations preserving integral operators [35]. Ali et al. [36] use the results obtained by Bulboacă [34] and give the sufficient conditions of certain normalized analytic functions f(z) to satisfy

$$\chi_{1}\left(z\right)≺\frac{f\left(z\right)}{zf^{\prime }\left(z\right)}≺\chi_{2}\left(z\right),$$

where $\chi_{1}$ and$\;\chi_{2}$ are univalent with $\chi_{1}\left(0\right)=\chi_{2}\left(0\right)=1$.

The following definitions and results are required in our work:

Definition 1.1 **[30]**

Denote Q by the subclass of H(U) and any f in Q is injective on $\bar{U}\setminus E\left(f\right)$, such that $E\left(f\right)=\left\{\zeta \in \partial U:\lim_{Z\to \zeta }f\left(z\right)=\infty \right\}$ and $f^{\prime }\left(\zeta \right)\neq 0$ for ζ∈∂U∖E(f). Further, let $Q_{a}$ denote the subclass of Q consisting of functions f for which f(0)=a.

Lemma 1.1 **[30]**

*Let* χ *be a univalent function from* U *into domain* D *,* Ξ *, and* Y *be analytic in a domain* D *with* Y *as non omitted value at* ω∈χ(U) *. Choose* $Q\left(z\right)=z\chi^{\prime }\left(z\right)Y\left(\chi (z)\right)$ *and* h(z)=Ξ(χ(z))+Q(z) *. Consider hypothetical circumstances under which*

(1) Q(z) is starlike and $Re(\frac{zh^{\prime }\left(z\right)}{Q(z)})>0$ for z∈U.
(2) ξ is analytic in U,ξ(U)⊂D,ξ(0)=χ(0), and
  (1.6) $$\Xi \left(\xi \left(z\right)\right)+z\xi^{\prime }\left(z\right)Y\left(\xi \left(z\right)\right)≺\Xi \left(\chi \left(z\right)\right)+z\chi^{\prime }\left(z\right)Y\left(\chi \left(z\right)\right).$$

Then ξ≺χ and the dominantχ of (1.6) is besting.

Lemma 1.2 **[34]**

*Let* χ *be a convex univalent function from* U *into domain* D *,* Ξ *and* Y *be analytic in a domain* D *. Consider hypothetical circumstances under with*

(1) $Q\left(z\right)=z\chi^{\prime }\left(z\right)Y\left(\chi (z)\right)$ is starlike and $Re(\frac{\Xi^{\prime }\left(\chi (z)\right)}{Y(\chi (z))})>0$, z∈U.
(2) ξ∈H[χ(0),1]∩Q, ξ(U)⊂D,where $\Xi \left(\xi (z)\right)+z\xi^{\prime }\left(z\right)Y\left(\xi (z)\right)$ is univalent in U and satisfying
  (1.7) $$\Xi \left(\chi \left(z\right)\right)+z\chi^{\prime }\left(z\right)Y\left(\chi \left(z\right)\right)≺\Xi \left(\xi \left(z\right)\right)+z\xi^{\prime }\left(z\right)Y\left(\xi \left(z\right)\right).$$

Then χ≺ξ and the dominantχ of (1.7) is besting.

Definition 1.2 **[37,38]**

Let Ω be a set in C, χ∈Q and $n\in Z^{+}$. The class of admissible functions $\Psi_{n}\;\left[\Omega ,\chi \right]$, consists of those functions $\psi :C^{3}\times U\to C$ that satisfy the admissibility condition:

(1.8) ψ(r,s,t;z)∉Ω

whenever

$$r=\chi \left(g\right),\;s=mg\chi^{\prime }\left(g\right),\;\text{and}$$

$$Re\left(\frac{t}{s}+1\right)\geq mRe\left(\frac{g\chi^{\prime \prime }\left(g\right)}{\chi^{\prime }\left(g\right)}+1\right)$$

z∈U,g∈∂U/E(χ),andm≥n.

Theorem 1.1 **[30]**

*Let*$\psi \in \Psi_{n}\;\left[\Omega ,\chi \right]$*with*χ(0)=a*, if*ξ∈H[a,n]*satisfies*

(1.9) $$\psi \left(\xi \left(z\right),\;z\xi^{\prime }\left(z\right),\;z^{2}\xi^{\prime \prime }\left(z\right);\;z\right)\in \Omega ,$$

then

ξ(z)≺χ(z).

Finally, our motivation is inspired by the significant contributions in the literature, particularly in works such as [6,8,18,36,[39], [40], [41], [42], [43], [44], [45]], which have explored the powerful potential of fractional calculus in various contexts. Building on these studies, we aim to leverage our new fractional operator to derive new and meaningful results in the areas of differential subordination and superordination. In this regard, we present several sandwich results that highlight the intricate interplay between fractional operators and analytic functions. Moreover, we introduce a new class of admissible functions that further extends the applicability of these concepts. Finally, motivated by the practical implications of fractional calculus, we explore its potential applications in fluid mechanics, demonstrating how our theoretical findings can contribute to the modeling and analysis of complex fluid flow behavior.

## (r,k)-Hypergeometric function and (r,k)-Srivastava fractional integral calculus

Firstly, it is pertinent to review the (r,k)−gamma function $\Gamma_{r,k}$, a generalization of ordinary gamma function, which was defined in [22] as follows, before introducing an extension of the hypergeometric function: for $z\in C,\;Re\left(z\right)>1-\frac{1}{r},\;r\in N,k>0$, then (r,k)−gamma function $\Gamma_{r,k}$ is defined by

(2.1) $$\Gamma_{r,k}\left(\delta \right)=\int_{0}^{\infty }t^{r\left(\delta -1\right)}\;e^{-\frac{t^{k}}{k}}\;dt.\;$$

Moreover,

(2.2) $$\Gamma_{r,k}\left(\delta \right)=k^{\frac{r\left(\delta -1\right)+1-k}{k}}\;\Gamma \left(\frac{r\left(\delta -1\right)+1}{k}\right).\;$$

In this section, we establish general results for well-known properties of hypergeometric functions pFq, including the concept of (r,k)-gamma function and Hadmard product. These results subsequently lead to the definition of a new fractional integral, which induces a new integral operator from $A_{p}$ into itself.

Definition 2.1 Let $a_{i}\in C,\;i=1,2,\ldots ,p,\;b_{j}\in C\setminus Z_{0}^{-},$ j=1,2,…,q,p≤q+1,k>0,r∈N. The (r,k)-hypergeometric function is defined by

(2.3) $$pF_{r,k}q\left(a_{1},\ldots ,a_{p};b_{1},\ldots b_{q};r,k;z\right)=\sum_{n=0}^{\infty }\frac{{\left(a_{1}\right)}_{n,r,k}\cdots {\left(a_{p}\right)}_{n,r,k}}{{\left(b_{1}\right)}_{n,r,k}\cdots {\left(b_{q}\right)}_{n,r,k}}\;\frac{z^{n}}{n!},$$

where ${\left(a\right)}_{n,r,k}$ is the (r,k)-Pochhammer is defined as below

$${\left(a\right)}_{n,r,k}=\frac{\Gamma_{r,k}\left(a+\frac{k}{r}n\right)}{\Gamma_{r,k}\left(a\right)\;},$$

equivalently,

$${\left(a\right)}_{n,r,k}=k^{n}{\left(\frac{r\left(a-1\right)+1}{k}\right)}_{n}.$$

**Fig. 1:**
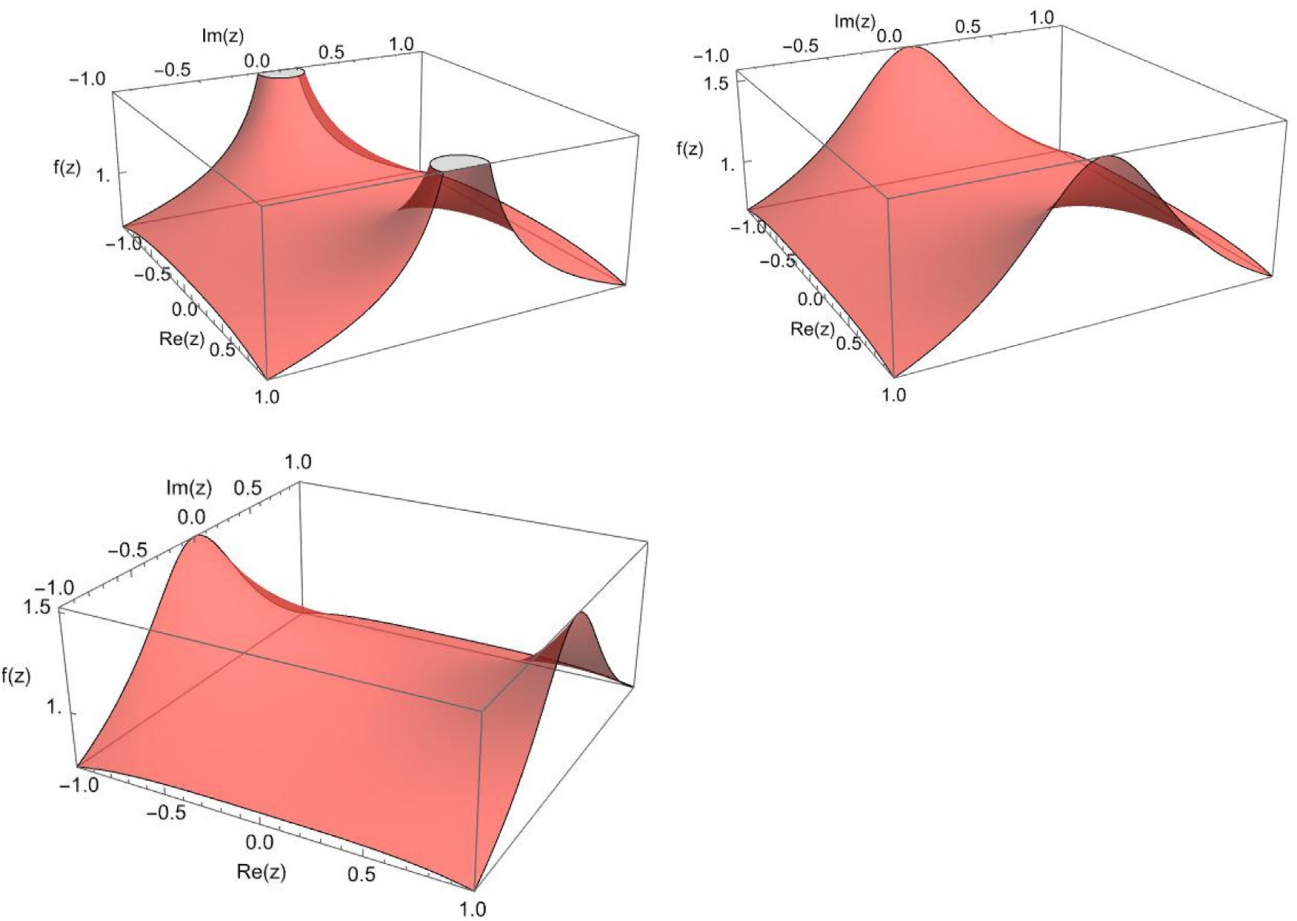
Complex plot of a,b, and c in Example 2.1 from −1−i to 1+i, resp.

Example 2.1 The following are some examples of Definition 2.1 (see Fig.1):

$$\left(a\right)\;2F_{1,1}1\left(0.5,0.5;0.75;1,1;z^{2}\right)=2F1\left(0.5,0.5;0.75;z^{2}\right)=\frac{\sin^{-1}\left(z\right)}{z}.$$

$$\left(b\right)\;2F_{1,0.5}1\left(0.5,0.5;0.75;1,0.5;z^{2}\right)=\sum_{n=0}^{\infty }\frac{{\left(0.5\right)}_{n,1,0.5}{\left(0.5\right)}_{n,1,0.5}}{{\left(0.75\right)}_{n,1,0.5}}\;\frac{z^{2n}}{n!}\;.$$

$$\left(c\right)\;2F_{2,0.5}1\left(0.75,0.75;0.9;2,0.5;z^{2}\right)=\sum_{n=0}^{\infty }\frac{{\left(0.75\right)}_{n,2,0.5}{\left(0.75\right)}_{n,2,0.5}}{{\left(0.9\right)}_{n,2,0.5}}\;\frac{z^{2n}}{n!}\;.$$

Theorem 2.1 *If*$Re\left(c-b\right)>1-\frac{1}{r},\;k\geq 1$*, then*

(2.4) $$2F_{r,k}1\left(a,b;c+\frac{1}{r}-1;r,k;z\right)*\left(kI\left(\frac{z}{k}\right)\right)=\frac{\Gamma_{r,k}\left(c+\frac{1}{r}-1\right)}{\Gamma_{r,k}\left(b\right)\Gamma_{r,k}\left(c-b\right)}\int_{0}^{1}t^{\frac{r\left(b-1\right)+1}{k}-1}\;{\left(1-t\right)}^{\frac{r\left(c-b-1\right)+1}{k}-1}\;{\left(1-tz\right)}^{-\frac{r\left(a-1\right)+1}{k}}dt,$$

where $I\left(z\right)=\frac{1}{1-z}$, arg(t)=arg(1−t)=0, and ${\left(1-tz\right)}^{-\frac{r(a-1)+1}{k}}$ has principle value.

**Proof.** By taking the right side of (2.4)

$$\frac{\Gamma_{r,k}\left(c+\frac{1}{r}-1\right)}{\Gamma_{r,k}\left(b\right)\Gamma_{r,k}\left(c-b\right)}\int_{0}^{1}t^{\frac{r\left(b-1\right)+1}{k}-1}\;{\left(1-t\right)}^{\frac{r\left(c-b-1\right)+1}{k}-1}\;{\left(1-tz\right)}^{-\frac{r\left(a-1\right)+1}{k}}\;dt$$

$$\;=\frac{\Gamma_{r,k}\left(c+\frac{1}{r}-1\right)}{\Gamma_{r,k}\left(b\right)\Gamma_{r,k}\left(c-b\right)}\;\sum_{n=0}^{\infty }\frac{{\left(\frac{r\left(a-1\right)+1}{k}\right)}_{n}\;z^{n}}{n!}\int_{0}^{1}t^{\frac{r\left(\frac{kn}{r}+b-1\right)+1}{k}-1}\;{\left(1-t\right)}^{\frac{r\left(c-b-1\right)+1}{k}-1}\;dt$$

$$\;=\frac{\Gamma_{r,k}\left(c+\frac{1}{r}-1\right)}{\Gamma_{r,k}\left(b\right)\Gamma_{r,k}\left(c-b\right)}\;\sum_{n=0}^{\infty }\frac{{\left(\frac{r\left(a-1\right)+1}{k}\right)}_{n}\;z^{n}}{n!}\left\{\;\frac{\Gamma \left(\frac{r\left(\frac{kn}{r}+b-1\right)+1}{k}\right)\;\Gamma \left(\frac{r\left(c-b-1\right)+1}{k}\right)}{\Gamma \left(\frac{r\left(\frac{kn}{r}+c-2\right)+2}{k}\right)}\right\}$$

$$\;=\frac{k^{\frac{r\left(c-2\right)+2}{k}-1}\;\Gamma \left(\frac{r\left(c-2\right)+2\;}{k}\right)\;}{k^{\frac{r\left(b-1\right)+1}{k}-1}\;\Gamma \left(\frac{r\left(b-1\right)+1}{k}\right)\;k^{\frac{r\left(c-b-1\right)+1}{k}-1}}\sum_{n=0}^{\infty }\frac{{\left(a\right)}_{n,r,k}\;z^{n}}{\;k^{n}\;n!}\left\{\;\frac{\Gamma \left(\frac{r\left(\frac{kn}{r}+b-1\right)+1}{k}\right)\;}{\Gamma \left(\frac{r\left(\frac{kn}{r}+c-2\right)+2}{k}\right)}\right\}$$

$$\;=k\sum_{n=0}^{\infty }\frac{{\left(a\right)}_{n,r,k}\;{\left(b\right)}_{n,r,k}}{k^{n}\;{\left(c+\frac{1}{r}-1\right)}_{n,r,k}}\;\frac{z^{n}}{n!}$$

$$\;=2F_{r,k}1\left(a,b;c+\frac{1}{r}-1;r,k;z\right)*\left(kI\left(\frac{z}{k}\right)\right).$$

Theorem 2.2 *Let* Re(c−b−a)>0 *,* $Re\left(c-b\right)>1-\frac{1}{r}$ *,* k≥1 *. Then*

(2.5) $$2F_{r,k}1\left(a,b;c+\frac{1}{r}-1;r,k;1\right)*\left(kI\left(\frac{1}{k}\right)\right)=\frac{\Gamma_{r,k}\left(c+\frac{1}{r}-1\right)\;\Gamma_{r,k}\left(c-b-a+1-\frac{1}{r}\right)}{\Gamma_{r,k}\left(c-b\right)\;\Gamma_{r,k}\left(c-a\right)}\;$$

**Proof.** From (2.4)

$$2F_{r,k}1\left(a,b;c+\frac{1}{r}-1;r,k;1\right)*\left(kI\left(\frac{1}{k}\right)\right)=\frac{\Gamma_{r,k}\left(c+\frac{1}{r}-1\right)}{\Gamma_{r,k}\left(b\right)\Gamma_{r,k}\left(c-b\right)}\int_{0}^{1}t^{\frac{r\left(b-1\right)+1}{k}-1}\;{\left(1-t\right)}^{\frac{r\left(c-b-a\right)}{k}-1}\;dt$$

$$\;=\frac{\Gamma_{r,k}\left(c+\frac{1}{r}-1\right)}{\Gamma_{r,k}\left(b\right)\Gamma_{r,k}\left(c-b\right)}\;\left\{\frac{\Gamma \left(\frac{r\left(b-1\right)+1}{k}\right)\;\Gamma \left(\frac{r\left[\left(c-b-a+1-\frac{1}{r}\right)-1\right]+1}{k}\right)}{\Gamma \left(\frac{r\left(c-a-1\right)+1}{k}\right)}\right\}$$

$$\;=\frac{\Gamma_{r,k}\left(c+\frac{1}{r}-1\right)}{k^{\frac{r\left(b-1\right)+1}{k}}\Gamma_{r,k}\left(c-b\right)}\;\times \;\frac{\;\Gamma \left(\frac{r\left[\left(c-b-a+1-\frac{1}{r}\right)-1\right]+1}{k}\right)}{\Gamma \left(\frac{r\left(c-a-1\right)+1}{k}\right)}$$

$$\;=\frac{\Gamma_{r,k}\left(c+\frac{1}{r}-1\right)\;\Gamma_{r,k}\left(c-b-a+1-\frac{1}{r}\right)}{\Gamma_{r,k}\left(c-b\right)\;\Gamma_{r,k}\left(c-a\right)}..$$

Theorem 2.3 *If* $Re\left(c-e\right)>1-\frac{1}{r}$ *,* k≥1 *, and* |arg(1−z)|<π *, then*

(2.6) $$\begin{array}{ccc} 2F_{r,k}1\left(a,b;c+\frac{1}{r}-1;r,k;z\right)*\left(kI\left(\frac{z}{k}\right)\right) & = & \frac{\Gamma_{r,k}\left(c+\frac{1}{r}-1\right)}{\Gamma_{r,k}\left(e\right)\Gamma_{r,k}\left(c-e\right)}\int_{0}^{1}t^{\frac{r\left(e-1\right)+1}{k}-1}{\left(1-t\right)}^{\frac{r\left(c-e-1\right)+1}{k}-1}\\ & & \left[\;2F_{r,k}1\left(a,b;e;r,k;tz\right)*I\left(\frac{z}{k}\right)\right]\;dt\;.\; \end{array}$$

**Proof.** From Theorem 2.1, integrating $1F_{r,k}0\left(a;-;r,k;tz\right)*I\left(\frac{z}{k}\right)$ after multiplying by distribution beta $t^{\frac{r(b-1)+1}{k}-1}\;{\left(1-t\right)}^{\frac{r(c-b-1)+1}{k}-1}$ gives $2F_{r,k}1\left(a,b;c+\frac{1}{r}-1;r,k;z\right)*\left(kI(\frac{z}{k})\right)$.

More generally, from proof of Theorem 2.1, we obtain

(2.7) $$\begin{array}{ccc} 3F_{r,k}2\left(a,b,d;e,c+\frac{1}{r}-1;r,k;z\right)*\left(kI\left(\frac{z}{k}\right)\right) & = & \frac{\Gamma_{r,k}\left(c+\frac{1}{r}-1\right)}{\Gamma_{r,k}\left(d\right)\Gamma_{r,k}\left(c-d\right)}\int_{0}^{1}t^{\frac{r\left(d-1\right)+1}{k}-1}\;{\left(1-t\right)}^{\frac{r\left(c-d-1\right)+1}{k}-1}\\ & & \left[\;2F_{r,k}1\left(a,b;e;r,k;tz\right)*I\left(\frac{z}{k}\right)\right]dt.\; \end{array}$$

Now, take d=e into (2.6), we conclude that

$$\begin{array}{ccc} 2F_{r,k}1\left(a,b;c+\frac{1}{r}-1;r,k;z\right)*\left(kI\left(\frac{z}{k}\right)\right) & = & \frac{\Gamma_{r,k}\left(c+\frac{1}{r}-1\right)}{\Gamma_{r,k}\left(e\right)\Gamma_{r,k}\left(c-e\right)}\int_{0}^{1}t^{\frac{r\left(e-1\right)+1}{k}-1}\;{\left(1-t\right)}^{\frac{r\left(c-e-1\right)+1}{k}-1}\\ & & \;\left[2F_{r,k}1\left(a,b;e;r,k;tz\right)*I\left(\frac{z}{k}\right)\right]\;dt.\; \end{array}$$

Definition 2.2 Let $\lambda >1-\frac{1}{r}$, δ,σ∈R. (r,k)-Srivastava fractional integral denoted by $I_{r,k}^{\lambda ,\delta ,\sigma }$ and defined as follows:

(2.8) $$I_{r,k}^{\lambda ,\delta ,\sigma }f\left(z\right)=\frac{z^{-\frac{r\left(\lambda +\delta -2\right)+2}{k}}}{\Gamma_{r,k}\left(\lambda \right)}\int_{0}^{z}{\left(z-\zeta \right)}^{\frac{r\left(\lambda -1\right)+1}{k}-1}\left[2F_{r,k}1\left(\lambda +\delta ,-\sigma ;\lambda ;1-\frac{\zeta }{z}\right)*I\left(\frac{1}{k}-\frac{\zeta }{kz}\right)\right]\;f\left(\zeta \right)d\zeta ,$$

where f(z) is an analytic function in a simple connected region of C containing 0 with order $f\left(z\right)=o\left({\left|z\right|}^{\epsilon }\right)$, z→0, and ϵ>max{δ−σ,0}−1.

The definition here given of a generalized fractional integral operator is more general than that of Srivastava at el. $I_{z}^{\lambda ,\delta ,\sigma }$ given in [23]. Also in [6] Morais and Zayed introduced the fractional integro-differential operator $I_{z}^{\lambda ,\delta ,\sigma }\left(m\right)$ as a generalization of Srivastava integral operator. We note that

(1) $I_{1,1}^{\lambda ,\delta ,\sigma }f\left(z\right)=I_{z}^{\lambda ,\delta ,\sigma }f\left(z\right)$.
(2) $I_{1,1}^{\lambda ,\delta ,\sigma }f^{\left(m\right)}\left(z\right)=I_{z}^{\lambda ,\delta ,\sigma }\left(m\right)f\left(z\right)$.

It is significant to not that many authors defined the operators having hypergeometric under integration and $D_{z}^{-\lambda }$ associated with a real-valued function (see [[46], [47], [48], [49], [50]]).

Lemma 2.1 *Let* $\lambda >1-\frac{1}{r}$ *,* $\frac{k\mu +k-1}{r}>\delta -\sigma -2+\frac{1}{r}$ *.Then*

(2.9) $$I_{r,k}^{\lambda ,\delta ,\sigma }z^{\mu }=\frac{\Gamma_{r,k}\left(\frac{k\mu +k-1}{r}+1\right)\;\Gamma_{r,k}\left(\frac{k\mu +k-1}{r}-\delta +\sigma +2-\frac{1}{r}\right)}{\Gamma_{r,k}\left(\frac{k\mu +k-1}{r}-\delta +1\right)\Gamma_{r,k}\left(\frac{k\mu +k-1}{r}+\lambda +\sigma +1\right)}\;z^{\mu -\frac{r\left(\delta -1\right)+1}{k}}.\;$$

**Proof.** In view (2.8)

$$I_{r,k}^{\lambda ,\delta ,\sigma }z^{\mu }=\frac{z^{-\frac{r\left(\lambda +\delta -2\right)+2}{k}}}{\Gamma_{r,k}\left(\lambda \right)}\int_{0}^{z}{\left(z-\zeta \right)}^{\frac{r\left(\lambda -1\right)+1}{k}-1}\left[2F_{r,k}1\left(\lambda +\delta ,-\sigma ;\lambda ;1-\frac{\zeta }{z}\right)*I\left(\frac{1}{k}-\frac{\zeta }{kz}\right)\right]\;\zeta^{\mu }d\zeta .$$

Set $t=1-\frac{\zeta }{z}$, we obtain

$$I_{r,k}^{\lambda ,\delta ,\sigma }z^{\mu }=\frac{z^{\mu -\frac{r\left(\delta -1\right)+1}{k}}}{\Gamma_{r,k}\left(\lambda \right)}\int_{0}^{1}t^{\frac{r\left(\lambda -1\right)+1}{k}-1}{\left(1-t\right)}^{\mu }\left[2F_{r,k}1\left(\lambda +\delta ,-\sigma ;\lambda ;t\right)*I\left(\frac{t}{k}\right)\right]\;\zeta^{\mu }d\zeta .$$

Applying Theorem 2.3 with e=λ, $c=\frac{k\mu +k-1}{r}+\lambda +1$, a=λ+δ, and b=−σ, we obtain

$$I_{r,k}^{\lambda ,\delta ,\sigma }z^{\mu }=\frac{z^{\mu -\frac{r\left(\delta -1\right)+1}{k}}}{\Gamma_{r,k}\left(\lambda \right)}\;\times \frac{\Gamma_{r,k}\left(\lambda \right)\;\Gamma_{r,k}\left(\frac{k\mu +k-1}{r}+1\right)}{\Gamma_{r,k}\left\{\left(\frac{k\mu +k-1}{r}+\lambda +1\right)+\frac{1}{r}-1\right\}}\left[2F_{r,k}1\left(\lambda +\delta ,-\sigma ;\lambda ;1\right)*\left(kI\left(\frac{1}{k}\right)\right)\right].$$

Theorem 2.2 follows

$$I_{r,k}^{\lambda ,\delta ,\sigma }z^{\mu }=\frac{\Gamma_{r,k}\left(\frac{k\mu +k-1}{r}+1\right)\;\Gamma_{r,k}\left(\frac{k\mu +k-1}{r}-\delta +\sigma +2-\frac{1}{r}\right)}{\Gamma_{r,k}\left(\frac{k\mu +k-1}{r}-\delta +1\right)\Gamma_{r,k}\left(\frac{k\mu +k-1}{r}+\lambda +\sigma +1\right)}\;z^{\mu -\frac{r\left(\delta -1\right)+1}{k}}.$$

Finally, we use above lemma to define $T_{r,k}^{\lambda ,\delta ,\sigma }:A_{p}\to A_{p}$ by

$$\begin{array}{ccc} T_{r,k}^{\lambda ,\delta ,\sigma }f\left(z\right) & = & \frac{\Gamma_{r,k}\left(\frac{kp+k-1}{r}-\delta +1\right)\Gamma_{r,k}\left(\frac{kp+k-1}{r}+\lambda +\sigma +1\right)}{\Gamma_{r,k}\left(\frac{kp+k-1}{r}+1\right)\;\Gamma_{r,k}\left(\frac{kp+k-1}{r}-\delta +\sigma +2-\frac{1}{r}\right)}z^{\frac{r\left(\delta -1\right)+1}{k}}I_{r,k}^{\lambda ,\delta ,\sigma }f\left(z\right)\\ & = & z^{p}+\sum_{n=p+1}^{\infty }\frac{\Gamma_{r,k}\left(\frac{kp+k-1}{r}-\delta +1\right)\Gamma_{r,k}\left(\frac{kp+k-1}{r}+\lambda +\sigma +1\right)\Gamma_{r,k}\left(\frac{kn+k-1}{r}+1\right)\;\Gamma_{r,k}\left(\frac{kn+k-1}{r}-\delta +\sigma +2-\frac{1}{r}\right)}{\Gamma_{r,k}\left(\frac{kp+k-1}{r}+1\right)\;\Gamma_{r,k}\left(\frac{kp+k-1}{r}-\delta +\sigma +2-\frac{1}{r}\right)\Gamma_{r,k}\left(\frac{kn+k-1}{r}-\delta +1\right)\Gamma_{r,k}\left(\frac{kn+k-1}{r}+\lambda +\sigma +1\right)}\;z^{n}. \end{array}$$

Easily to cheek that

(2.10) $$z{\left(T_{r,k}^{\lambda ,\delta ,\sigma }f\left(z\right)\right)}^{\prime }=\left[p+\frac{r}{k}\left(\lambda +\sigma \right)\right]T_{r,k}^{\lambda -\frac{k}{r},\delta ,\sigma }f\left(z\right)-\frac{r}{k}\left(\lambda +\sigma \right)T_{r,k}^{\lambda ,\delta ,\sigma }f\left(z\right),\;$$

where $r\in N,\;k\in R^{+},\;\delta ,\sigma \in R$, and $\lambda >1-\frac{1-k}{r}$.

In special case

$$T_{z}^{\lambda ,\delta ,\sigma }f\left(z\right)=T_{1,1}^{\lambda ,\delta ,\sigma }f\left(z\right)=z^{p}+\sum_{n=p+1}^{\infty }\frac{\Gamma \left(p-\delta +1\right)\Gamma \left(p+\lambda +\sigma +1\right)\Gamma \left(n+1\right)\;\Gamma \left(n-\delta +\sigma +1\right)}{\Gamma \left(p+1\right)\;\Gamma \left(p-\delta +\sigma +1\right)\Gamma \left(n-\delta +1\right)\Gamma \left(n+\lambda +\sigma +1\right)}\;z^{n},$$

and

(2.11) $$z{\left(T_{z}^{\lambda ,\delta ,\sigma }f\left(z\right)\right)}^{\prime }=\left(p+\lambda +\sigma \right)T_{z}^{\lambda -1,\delta ,\sigma }f\left(z\right)-\left(\lambda +\sigma \right)T_{z}^{\lambda ,\delta ,\sigma }f\left(z\right).$$

## Differential subordination results

Unless indicated otherwise, we will assume throughout the rest of this paper that$\;r\in N,\;k\in R^{+},\;\delta ,\sigma \in R$, and $\lambda >1-\frac{1-2k}{r}$. In this section, we present applications of differential subordination and superordination, incorporating our fractional operator. The applications lead to the presentation of corollaries and remarks, some of which generalize well-known results, such as Marx-Strohhaker theorem.

Theorem 3.1 *Let*χ*be univalent in*U*with*χ(0)=1*,*$\varphi ,\varrho \in A_{p}$*,*$\theta ,\omega \in R^{+}$*and,*$\gamma \in R^{*}$. Also, suppose that$\frac{z\chi^{\prime }\left(z\right)}{\chi (z)}$*is starlike and*Re(χ(z))>0*.If*$f\in A_{p}$*satisfies the below inequality:*

(3.1) $$\Omega_{1}\left(z\right)≺1+\theta \chi \left(z\right)+\frac{z\omega \chi^{\prime }\left(z\right)}{\chi \left(z\right)},$$

where

(3.2) $$\Omega_{1}\left(z\right)=1+\theta {\left(\frac{T_{r,k}^{\lambda -\frac{k}{r},\delta ,\sigma }\left(f*\varphi \right)\left(z\right)}{T_{r,k}^{\lambda ,\delta ,\sigma }\left(f*\varrho \right)\left(z\right)}\right)}^{\gamma }+\gamma \omega \left[\left(p+\frac{r}{k}\left(\lambda +\sigma \right)-1\right)\frac{T_{r,k}^{\lambda -\frac{2k}{r},\delta ,\sigma }\left(f*\varphi \right)\left(z\right)}{T_{r,k}^{\lambda -\frac{k}{r},\delta ,\sigma }\left(f*\varphi \right)\left(z\right)}-\left(p+\frac{r}{k}\left(\lambda +\sigma \right)\right)\frac{T_{r,k}^{\lambda -\frac{k}{r},\delta ,\sigma }\left(f*\varrho \right)\left(z\right)}{T_{r,k}^{\lambda ,\delta ,\sigma }\left(f*\varrho \right)\left(z\right)}+1\right],$$

then

$${\left(\frac{T_{r,k}^{\lambda -\frac{k}{r},\delta ,\sigma }\left(f*\varphi \right)\left(z\right)}{T_{r,k}^{\lambda ,\delta ,\sigma }\left(f*\varrho \right)\left(z\right)}\right)}^{\gamma }≺\chi \left(z\right),$$

and χ(z) is the best dominant of (3.1).

**Proof.** Define

(3.3) $$\xi \left(z\right)={\left(\frac{T_{r,k}^{\lambda -\frac{k}{r},\delta ,\sigma }\left(f*\varphi \right)\left(z\right)}{T_{r,k}^{\lambda ,\delta ,\sigma }\left(f*\varrho \right)\left(z\right)}\right)}^{\gamma },\;z\in U.$$

Then ξ is an analytic and ξ(0)=1. After some computation, we get

(3.4) $$\frac{z\xi^{\prime }\left(z\right)}{\xi \left(z\right)}=\gamma \left(\frac{z{\left(T_{r,k}^{\lambda -\frac{k}{r},\delta ,\sigma }\left(f*\varphi \right)\left(z\right)\right)}^{\prime }}{T_{r,k}^{\lambda -\frac{k}{r},\delta ,\sigma }\left(f*\varrho \right)\left(z\right)}-\frac{z{\left(T_{r,k}^{\lambda ,\delta ,\sigma }\left(f*\varphi \right)\left(z\right)\right)}^{\prime }}{T_{r,k}^{\lambda ,\delta ,\sigma }\left(f*\varrho \right)\left(z\right)}\right).$$

Substituting (2.10) into (3.4), we obtain

$$\frac{z\xi^{\prime }\left(z\right)}{\xi \left(z\right)}=\gamma \left[\left(p+\frac{r}{k}\left(\lambda +\sigma \right)-1\right)\frac{T_{r,k}^{\lambda -\frac{2k}{r},\delta ,\sigma }\left(f*\varphi \right)\left(z\right)}{T_{r,k}^{\lambda -\frac{k}{r},\delta ,\sigma }\left(f*\varphi \right)\left(z\right)}-\left(p+\frac{r}{k}\left(\lambda +\sigma \right)\right)\frac{T_{r,k}^{\lambda -\frac{k}{r},\delta ,\sigma }\left(f*\varrho \right)\left(z\right)}{T_{r,k}^{\lambda ,\delta ,\sigma }\left(f*\varrho \right)\left(z\right)}+1\right].$$

Consequently,

(3.5) $$1+\theta \xi \left(z\right)+\frac{z\omega \xi^{\prime }\left(z\right)}{\xi \left(z\right)}=\Omega_{1}\left(z\right),$$

where $\Omega_{1}$ is given by (3.2).

Using (3.1) and (3.5), we obtain

$$1+\theta \xi \left(z\right)+\frac{z\omega \xi^{\prime }\left(z\right)}{\xi \left(z\right)}≺1+\theta \chi \left(z\right)+\frac{z\omega \chi^{\prime }\left(z\right)}{\chi \left(z\right)}.$$

By putting Ξ(w)=1+θw and $Y\left(w\right)=\frac{\omega }{w}$. Obviously, cheek that Ξ(w) and Y(w) are analytic in C∖{0} and that Y(w)≠0, w∈C∖{0}. We obtain

$$Q\left(z\right)=z\chi^{\prime }\left(z\right)Y\left(\chi \left(z\right)\right)=\frac{z\omega \chi^{\prime }\left(z\right)}{\chi \left(z\right)},$$

and

$$h\left(z\right)=\Xi \left(\chi \left(z\right)\right)+Q\left(z\right)=1+\;\theta \chi \left(z\right)+\frac{z\omega \chi^{\prime }\left(z\right)}{\chi \left(z\right)}.$$

In light of the hypothesis, it is observed that Q(z) is starlike and

$$Re\left\{\frac{zh^{\prime }\left(z\right)}{Q\left(z\right)}\right\}=Re\left\{\frac{z\left(\;\theta \chi^{\prime }\left(z\right)+Q^{\prime }\left(z\right)\right)}{Q\left(z\right)}\right\}=Re\left\{\frac{\theta }{\omega }\chi \left(z\right)\right\}+Re\left\{\frac{zQ^{\prime }\left(z\right)}{Q\left(z\right)}\right\}>0.$$

Thus Lemma 1.1 leads to requirement .

Choose $\varphi \left(z\right)=\varrho \left(z\right)=z^{p}/1-z$ and χ(z)=1+z/1−z, then Re(χ(z))>0 and $Q\left(z\right)=z\chi^{\prime }\left(z\right)/\chi \left(z\right)$ is starlike. Taking ω=p=r=k=1 into Theorem 3.1, we have the following corollary:

Corollary 3.1 *Let*f∈A*,*θ>0*, and*$\gamma \in R^{*}$. If

$$1+\theta {\left(\frac{T_{z}^{\lambda -1,\delta ,\sigma }f\left(z\right)}{T_{z}^{\lambda ,\delta ,\sigma }f\left(z\right)}\right)}^{\gamma }+\gamma \left[\left(\lambda +\sigma \right)\frac{T_{z}^{\lambda -2,\delta ,\sigma }f\left(z\right)}{T_{z}^{\lambda -1,\delta ,\sigma }f\left(z\right)}-\left(1+\lambda +\sigma \right)\frac{T_{z}^{\lambda -1,\delta ,\sigma }f\left(z\right)}{T_{z}^{\lambda ,\delta ,\sigma }f\left(z\right)}+1\right]≺1+\theta \frac{1+z}{1-z}+\frac{2z}{1-z^{2}},$$

then

$${\left(\frac{T_{z}^{\lambda -1,\delta ,\sigma }f\left(z\right)}{T_{z}^{\lambda ,\delta ,\sigma }f\left(z\right)}\right)}^{\gamma }≺\frac{1+z}{1-z},$$

or equivalently,

$$Re\left({\left(\frac{T_{z}^{\lambda -1,\delta ,\sigma }f\left(z\right)}{T_{z}^{\lambda ,\delta ,\sigma }f\left(z\right)}\right)}^{\gamma }\right)>0.$$

Choose $\varphi \left(z\right)=\varrho \left(z\right)=z^{p}/1-z$ and $\chi \left(z\right)=e^{z}$, then Re(χ(z))>0 and $Q\left(z\right)=z\chi^{\prime }\left(z\right)/\chi \left(z\right)=z$ is starlike. Taking ω=p=r=k=1 into Theorem 3.1, we have the following corollary:

Corollary 3.2 *Let*f∈A*,*θ>0*, and*$\gamma \in R^{*}$. If

$$1+\theta {\left(\frac{T_{z}^{\lambda -1,\delta ,\sigma }f\left(z\right)}{T_{z}^{\lambda ,\delta ,\sigma }f\left(z\right)}\right)}^{\gamma }+\gamma \left[\left(\lambda +\sigma \right)\frac{T_{z}^{\lambda -2,\delta ,\sigma }f\left(z\right)}{T_{z}^{\lambda -1,\delta ,\sigma }f\left(z\right)}-\left(1+\lambda +\sigma \right)\frac{T_{z}^{\lambda -1,\delta ,\sigma }f\left(z\right)}{T_{z}^{\lambda ,\delta ,\sigma }f\left(z\right)}+1\right]≺1+z+\theta e^{z},$$

then

$${\left(\frac{T_{z}^{\lambda -1,\delta ,\sigma }f\left(z\right)}{T_{z}^{\lambda ,\delta ,\sigma }f\left(z\right)}\right)}^{\gamma }≺e^{z}.$$

Choose $\varphi \left(z\right)=\varrho \left(z\right)=z^{p}/1-z$ and χ(z)=1/1−z, then Re(χ(z))>0 and $Q\left(z\right)=z\chi^{\prime }\left(z\right)/\chi \left(z\right)=z/1-z$ is starlike. Taking ω=p=r=k=1 and θ=0 into Theorem 3.1, we have the following corollary:

Corollary 3.3 *Let*f∈A*,* *and*$\gamma \in R^{*}$. If

$$Re\left(1+\gamma \left[\left(\lambda +\sigma \right)\frac{T_{z}^{\lambda -2,\delta ,\sigma }f\left(z\right)}{T_{z}^{\lambda -1,\delta ,\sigma }f\left(z\right)}-\left(1+\lambda +\sigma \right)\frac{T_{z}^{\lambda -1,\delta ,\sigma }f\left(z\right)}{T_{z}^{\lambda ,\delta ,\sigma }f\left(z\right)}+1\right]\right)>\frac{1}{2},$$

then

$$Re\left({\left(\frac{T_{z}^{\lambda -1,\delta ,\sigma }f\left(z\right)}{T_{z}^{\lambda ,\delta ,\sigma }f\left(z\right)}\right)}^{\gamma }\right)>\frac{1}{2}\;.$$

Theorem 3.2 *Let*χ*be univalent in*U*with*χ(0)=1*,*$\varphi ,\varrho \in A_{p}$*,*$\theta ,\omega \in R^{+}$*,*$l_{1},l_{2}\in C$*with*$l_{1}+l_{2}\neq 0$*, and*$\gamma \in R^{*}$. Also, suppose that$\frac{z\chi^{\prime }\left(z\right)}{\chi (z)}$*is starlike and*Re(χ(z))>0*.If*$f\in A_{p}$*satisfies the below inequality:*

(3.6) $$\Omega_{2}\left(z\right)≺1+\theta \chi \left(z\right)+\frac{z\omega \chi^{\prime }\left(z\right)}{\chi \left(z\right)},\;$$

where

(3.7) $$\begin{array}{ccc} \Omega_{2}\left(z\right) & = & 1+\theta {\left(\frac{l_{1}T_{r,k}^{\lambda -\frac{k}{r},\delta ,\sigma }\left(f*\varphi \right)\left(z\right)+l_{2}T_{r,k}^{\lambda ,\delta ,\sigma }\left(f*\varrho \right)\left(z\right)\;}{\left(l_{1}+l_{2}\right)z^{p}}\right)}^{\gamma }\\ & & +\;\gamma \omega \left[\frac{l_{1}\left(p+\frac{r}{k}\left(\lambda +\sigma \right)-1\right)\left(T_{r,k}^{\lambda -\frac{2k}{r},\delta ,\sigma }\left(f*\varphi \right)\left(z\right)-T_{r,k}^{\lambda -\frac{k}{r},\delta ,\sigma }\left(f*\varphi \right)\left(z\right)\right)+l_{2}\left(p+\frac{r}{k}\left(\lambda +\sigma \right)\right)\left(T_{r,k}^{\lambda -\frac{k}{r},\delta ,\sigma }\left(f*\varrho \right)\left(z\right)-T_{r,k}^{\lambda ,\delta ,\sigma }\left(f*\varrho \right)\left(z\right)\right)}{l_{1}T_{r,k}^{\lambda -\frac{k}{r},\delta ,\sigma }\left(f*\varphi \right)\left(z\right)+l_{2}T_{r,k}^{\lambda ,\delta ,\sigma }\left(f*\varrho \right)\left(z\right)}\right], \end{array}$$

then

$${\left(\frac{l_{1}T_{r,k}^{\lambda -\frac{k}{r},\delta ,\sigma }\left(f*\varphi \right)\left(z\right)+l_{2}T_{r,k}^{\lambda ,\delta ,\sigma }\left(f*\varrho \right)\left(z\right)\;}{\left(l_{1}+l_{2}\right)z^{p}}\right)}^{\gamma }≺\chi \left(z\right),$$

and χ(z) is the best dominant of (3.9).

**Proof.** Define

(3.8) $$\xi \left(z\right)={\left(\frac{l_{1}T_{r,k}^{\lambda -\frac{k}{r},\delta ,\sigma }\left(f*\varphi \right)\left(z\right)+l_{2}T_{r,k}^{\lambda ,\delta ,\sigma }\left(f*\varrho \right)\left(z\right)\;}{\left(l_{1}+l_{2}\right)z^{p}}\right)}^{\gamma },\;\left(z\in U\right).$$

Then the function ξ(z) is analytic and ξ(0)=1.

After direct computation, we obtain

(3.9) $$\frac{z\xi^{\prime }\left(z\right)}{\xi \left(z\right)}=\gamma \left[\frac{l_{1}\left(p+\frac{r}{k}\left(\lambda +\sigma \right)-1\right)\left(T_{r,k}^{\lambda -\frac{2k}{r},\delta ,\sigma }\left(f*\varphi \right)\left(z\right)-T_{r,k}^{\lambda -\frac{k}{r},\delta ,\sigma }\left(f*\varphi \right)\left(z\right)\right)+l_{2}\left(p+\frac{r}{k}\left(\lambda +\sigma \right)\right)\left(T_{r,k}^{\lambda -\frac{k}{r},\delta ,\sigma }\left(f*\varrho \right)\left(z\right)-T_{r,k}^{\lambda ,\delta ,\sigma }\left(f*\varrho \right)\left(z\right)\right)}{l_{1}T_{r,k}^{\lambda -\frac{k}{r},\delta ,\sigma }\left(f*\varphi \right)\left(z\right)+l_{2}T_{r,k}^{\lambda ,\delta ,\sigma }\left(f*\varrho \right)\left(z\right)}\right].$$

Consequently,

(3.10) $$\Omega_{2}\left(z\right)=1+\theta \xi \left(z\right)+\frac{z\omega \xi^{\prime }\left(z\right)}{\xi \left(z\right)}.\;$$

Substituting (3.10) into (3.6), we have

$$1+\theta \xi \left(z\right)+\frac{z\omega \xi^{\prime }\left(z\right)}{\xi \left(z\right)}≺1+\theta \chi \left(z\right)+\frac{z\omega \chi^{\prime }\left(z\right)}{\chi \left(z\right)}.$$

By choose Ξ(w)=1+θw, and $Y\left(w\right)=\frac{\omega }{w}$ . Obviously, cheek that Ξ(w) and Y(w) are analytic in C∖{0} and that Y(w)≠0, w∈C∖{0}. We obtain

$$Q\left(z\right)=z\chi^{\prime }\left(z\right)Y\left(\chi \left(z\right)\right)=\frac{z\omega \chi^{\prime }\left(z\right)}{\chi \left(z\right)},$$

and

$$h\left(z\right)=\Xi \left(\chi \left(z\right)\right)+Q\left(z\right)=1+\;\theta \chi \left(z\right)+\frac{z\omega \chi^{\prime }\left(z\right)}{\chi \left(z\right)}\;.$$

In light of the hypothesis, it is observed that Q(z) is starlike and

$$Re\left\{\frac{zh^{\prime }\left(z\right)}{Q\left(z\right)}\right\}=Re\left\{\frac{z\left(\;\theta \chi^{\prime }\left(z\right)+Q^{\prime }\left(z\right)\right)}{Q\left(z\right)}\right\}=Re\left\{\frac{\theta }{\omega }\chi \left(z\right)\right\}+Re\left\{\frac{zQ^{\prime }\left(z\right)}{Q\left(z\right)}\right\}>0\;.$$

Hence, the proof is complete from applying Lemma 1.1.

Using the same substitutions mentioned in Corollary 3.1, 3.2, and 3.3, respectively, also select $l_{1}=0$, and without loss of generality we take λ>1. Consequently, we can directly obtain the following three corollaries from Theorem 3.2:

Corollary 3.4 *Let*f∈A*,*θ>0*, and*$\gamma \in R^{*}$. If

$$1+\theta {\left(\frac{T_{z}^{\lambda ,\delta ,\sigma }f\left(z\right)\;}{z}\right)}^{\gamma }+\gamma \left[\frac{\left(1+\lambda +\sigma \right)T_{z}^{\lambda -1,\delta ,\sigma }f\left(z\right)}{T_{r,k}^{\lambda ,\delta ,\sigma }f\left(z\right)}-\left(1+\lambda +\sigma \right)\right]≺1+\theta \frac{1+z}{1-z}+\frac{2z}{1-z^{2}},$$

then

$${\left(\frac{T_{z}^{\lambda ,\delta ,\sigma }f\left(z\right)\;}{z}\right)}^{\gamma }≺\frac{1+z}{1-z},$$

or equivalently,

$$Re\left({\left(\frac{T_{z}^{\lambda ,\delta ,\sigma }f\left(z\right)\;}{z}\right)}^{\gamma }\right)>0.$$

Corollary 3.5 *Let*f∈A*,*θ>0*, and*$\gamma \in R^{*}$. If

$$1+\theta {\left(\frac{T_{z}^{\lambda ,\delta ,\sigma }f\left(z\right)\;}{z}\right)}^{\gamma }+\;\gamma \left[\frac{\left(1+\lambda +\sigma \right)T_{z}^{\lambda -1,\delta ,\sigma }f\left(z\right)}{T_{r,k}^{\lambda ,\delta ,\sigma }f\left(z\right)}-\left(1+\lambda +\sigma \right)\right]≺1+z+\theta e^{z},$$

then

$${\left(\frac{T_{z}^{\lambda ,\delta ,\sigma }f\left(z\right)\;}{z}\right)}^{\gamma }≺e^{z}.$$

Corollary 3.6 *Let*f∈A*,* *and*$\gamma \in R^{*}$. If

$$Re\left(1+\;\gamma \left[\frac{\left(1+\lambda +\sigma \right)T_{z}^{\lambda -1,\delta ,\sigma }f\left(z\right)}{T_{r,k}^{\lambda ,\delta ,\sigma }f\left(z\right)}-\left(1+\lambda +\sigma \right)\right]\right)>\frac{1}{2},$$

then

$$Re\left({\left(\frac{T_{z}^{\lambda ,\delta ,\sigma }f\left(z\right)\;}{z}\right)}^{\gamma }\right)>\frac{1}{2}\;.$$

Remark 3.1 Let f∈A. Put $zf^{\prime }\left(z\right)=g\left(z\right)$, then g∈A and

$$f\left(z\right)=\int_{0}^{z}\frac{g\left(w\right)}{w}dw.$$

Choose, λ−1→0, δ=1−λ, σ=−1, γ=1, then

(3.11) $$T_{z}^{\lambda -1,\delta ,\sigma }g\left(z\right)=g\left(z\right).$$

On the other hand,

(3.12) $$T_{z}^{\lambda ,\delta ,\sigma }g\left(z\right)=\int_{0}^{z}\frac{g\left(w\right)}{w}dw.$$

Consequently, it can be concluded that Corollary 3.6 generalizes one of the conditions for Marx-Strohhacker theorem (see [51,52])

Theorem 3.3 *Let* χ *be convex in* U *with* χ(0)=1 *,* $\varphi ,\varrho \in A_{p}$ *,* $\theta ,\omega \in R^{+}$ *and,* $\gamma \in R^{*}$ *. Also, suppose that* $\frac{z\chi^{\prime }\left(z\right)}{\chi (z)}$ *is starlike and* ${\left(\chi^{2}\right)}^{\prime }\left(z\right)$ *has positive real part in* U *, respectively .If* $f\in A_{p}$ *and*

$${\left(\frac{T_{r,k}^{\lambda -\frac{k}{r},\delta ,\sigma }\left(f*\varphi \right)\left(z\right)}{T_{r,k}^{\lambda ,\delta ,\sigma }\left(f*\varrho \right)\left(z\right)}\right)}^{\gamma }\in H\left[1,1\right]\cap Q,$$

$\Omega_{1}\left(z\right)$ is given by (3.2), and $\Omega_{1}$ is an univalent in U. If

(3.13) $$\rho +\theta \chi \left(z\right)+\frac{\omega z\chi^{\prime }\left(z\right)}{\chi \left(z\right)}≺\Omega_{1}\left(z\right),\;$$

then

$$\chi \left(z\right)≺{\left(\frac{T_{r,k}^{\lambda -\frac{k}{r},\delta ,\sigma }\left(f*\varphi \right)\left(z\right)}{T_{r,k}^{\lambda ,\delta ,\sigma }\left(f*\varrho \right)\left(z\right)}\right)}^{\gamma },$$

and χ(z) is the best dominant of (3.13).

**Proof.** Define

$$\xi \left(z\right)={\left(\frac{T_{r,k}^{\lambda -\frac{k}{r},\delta ,\sigma }\left(f*\varphi \right)\left(z\right)}{T_{r,k}^{\lambda ,\delta ,\sigma }\left(f*\varrho \right)\left(z\right)}\right)}^{\gamma },\;z\in U.$$

Then ξ is an analytic and ξ(0)=1. It follows from (2.10), (3.2), and (3.13) that

$$\rho +\theta \chi \left(z\right)+\frac{\omega z\chi^{\prime }\left(z\right)}{\chi \left(z\right)}≺\rho +\theta \xi \left(z\right)+\frac{\omega z\xi^{\prime }\left(z\right)}{\chi \left(z\right)}.$$

By choose Ξ(w)=1+θw and $Y\left(w\right)=\frac{\omega }{w}$ . Obviously, the cheek that Ξ(w) and Y(w) are analytic in C∖{0} and that Y(w)≠0, w∈C∖{0}. Since ${\left(\chi^{2}\right)}^{\prime }\left(z\right)$ has positive real part, then

$$Re\left\{\frac{\Xi^{\prime }\left(\chi \left(z\right)\right)}{Y\left(\chi \left(z\right)\right)}\right\}=Re\left\{\frac{\theta }{\omega }\chi \left(z\right)\chi^{\prime }\left(z\right)\right\}>0,$$

also from hypothesis Q(z) is starlike The application of Lemma 1.2 leads to the requirement.

Let χ be convex in U with χ(0)=1, $\varphi ,\varrho \in A_{p}$, $\theta ,\omega \in R^{+}$and,$\gamma \in R^{*}$. Also, suppose that $\frac{z\chi^{\prime }\left(z\right)}{\chi (z)}$ and ${\left(\chi^{2}\right)}^{\prime }\left(z\right)$ are starlike and univalent close-to-convex in U, respectively .If $f\in A_{p}$ and

$${\left(\frac{T_{r,k}^{\lambda -\frac{k}{r},\delta ,\sigma }\left(f*\varphi \right)\left(z\right)}{T_{r,k}^{\lambda ,\delta ,\sigma }\left(f*\varrho \right)\left(z\right)}\right)}^{\gamma }\in H\left[1,1\right]\cap Q,$$

$\Omega_{1}\left(z\right)$ is given by (3.3), and $\Omega_{1}$ is an univalent in U. If

(3.14) $$\rho +\theta \chi \left(z\right)+\frac{\omega z\chi^{\prime }\left(z\right)}{\chi \left(z\right)}≺\Omega_{1}\left(z\right),\;$$

then

$$\chi \left(z\right)≺{\left(\frac{T_{r,k}^{\lambda -\frac{k}{r},\delta ,\sigma }\left(f*\varphi \right)\left(z\right)}{T_{r,k}^{\lambda ,\delta ,\sigma }\left(f*\varrho \right)\left(z\right)}\right)}^{\gamma },$$

and χ(z) is the best dominant of (3.13).

Theorem 3.4 *Let*χ*be convex in*U*with*χ(0)=1*,*$\varphi ,\varrho \in A_{p}$*,*$\theta ,\omega \in R^{+}$*and,*$\gamma \in R^{*}$. Also, suppose that$\frac{z\chi^{\prime }\left(z\right)}{\chi (z)}$*is starlike and*${\left(\chi^{2}\right)}^{\prime }\left(z\right)$*has positive real part in*U*, respectively .If*$f\in A_{p}$*and*

$${\left(\frac{l_{1}T_{r,k}^{\lambda -\frac{k}{r},\delta ,\sigma }\left(f*\varphi \right)\left(z\right)+l_{2}T_{r,k}^{\lambda ,\delta ,\sigma }\left(f*\varrho \right)\left(z\right)\;}{\left(l_{1}+l_{2}\right)z^{p}}\right)}^{\gamma }\in H\left[1,1\right]\cap Q,$$

where $\Omega_{2}\left(z\right)$ is given by (3.7), and $\Omega_{2}$ is an univalent in U. If

(3.15) $$\rho +\theta \chi \left(z\right)+\frac{\omega z\chi^{\prime }\left(z\right)}{\chi \left(z\right)}≺\Omega_{2}\left(z\right),\;$$

then

$$\chi \left(z\right)≺{\left(\frac{l_{1}T_{r,k}^{\lambda -\frac{k}{r},\delta ,\sigma }\left(f*\varphi \right)\left(z\right)+l_{2}T_{r,k}^{\lambda ,\delta ,\sigma }\left(f*\varrho \right)\left(z\right)\;}{\left(l_{1}+l_{2}\right)z^{p}}\right)}^{\gamma },$$

and χ(z) is the best dominant of (3.15).

**Proof.** The result is followed by applying the same method as Theorem 3.3, setting

$$\xi \left(z\right)={\left(\frac{l_{1}T_{r,k}^{\lambda -\frac{k}{r},\delta ,\sigma }\left(f*\varphi \right)\left(z\right)+l_{2}T_{r,k}^{\lambda ,\delta ,\sigma }\left(f*\varrho \right)\left(z\right)\;}{\left(l_{1}+l_{2}\right)z^{p}}\right)}^{\gamma }.$$

## Sandwich results

Combining Theorems 3.1, 3.2, 3.3, and 3.4, we conclude the following sandwich results for p-valent functions involving $T_{r,k}^{\lambda ,\delta ,\sigma }$:

Theorem 4.1 *Let* $\chi_{1}$ *and* $\chi_{2}$ *be convex univalent functions in* U *with* $\chi_{1}\left(0\right)=\chi_{2}\left(0\right)=1$ *,* $\varphi ,\varrho \in A_{p}$ *,* $\theta ,\omega \in R^{+}$ *and,* $\gamma \in R^{*}$ *. Moreover,* $z{\chi_{1}}^{\prime }\left(z\right)/\chi_{1}\left(z\right)$ *and* $z{\chi_{2}}^{\prime }\left(z\right)/\chi_{2}\left(z\right)$ *are starlike in* U *, also suppose the real part of* $\chi_{2}\left(z\right)$ *and* ${\left({\chi_{1}}^{2}\right)}^{\prime }\left(z\right)$ *are positive .If* $f\in A_{p}$ *and*

$${\left(\frac{T_{r,k}^{\lambda -\frac{k}{r},\delta ,\sigma }\left(f*\varphi \right)\left(z\right)}{T_{r,k}^{\lambda ,\delta ,\sigma }\left(f*\varrho \right)\left(z\right)}\right)}^{\gamma }\in H\left[1,1\right]\cap Q,$$

$\Omega_{1}$ is given by (3.2), and $\Omega_{1}$ is an univalent in U. If

$$1+\theta \chi_{1}\left(z\right)+\frac{\omega z{\chi_{1}}^{\prime }\left(z\right)}{\chi_{1}\left(z\right)}≺\Omega_{1}\left(z\right)\;≺\;1+\theta \chi_{2}\left(z\right)+\frac{\omega z{\chi_{2}}^{\prime }\left(z\right)}{\chi_{2}\left(z\right)},$$

then

$$\chi_{1}\left(z\right)≺{\left(\frac{T_{r,k}^{\lambda -\frac{k}{r},\delta ,\sigma }\left(f*\varphi \right)\left(z\right)}{T_{r,k}^{\lambda ,\delta ,\sigma }\left(f*\varrho \right)\left(z\right)}\right)}^{\gamma }≺\chi_{2}\left(z\right),$$

and the dominant $\chi_{2}$ (the subordinant $\chi_{1}$) is besting.

Theorem 3.2 *Let*$\chi_{1}$*and*$\chi_{2}$*be convex univalent functions in*U*with*$\chi_{1}\left(0\right)=\chi_{2}\left(0\right)=1$*,*$\varphi ,\varrho \in A_{p}$*,*$\theta ,\omega \in R^{+}$*,*$l_{1},l_{2}\in C$*with*$l_{1}+l_{2}\neq 0$*, and*$\gamma \in R^{*}$. Moreover,$\frac{z{\chi_{1}}^{\prime }\left(z\right)}{\chi_{1}\left(z\right)}$*and*$\frac{z{\chi_{2}}^{\prime }\left(z\right)}{\chi_{2}\left(z\right)}$*are starlike in*U*, also suppose the real parts of*$\chi_{2}\left(z\right)$*and*${\left({\chi_{1}}^{2}\right)}^{\prime }\left(z\right)$*are positive .If*$f\in A_{p}$*and*

$${\left(\frac{l_{1}T_{r,k}^{\lambda -\frac{k}{r},\delta ,\sigma }\left(f*\varphi \right)\left(z\right)+l_{2}T_{r,k}^{\lambda ,\delta ,\sigma }\left(f*\varrho \right)\left(z\right)\;}{\left(l_{1}+l_{2}\right)z^{p}}\right)}^{\gamma }\in H\left[1,1\right]\cap Q,$$

and $\Omega_{2}$ is given by (3.7). If $\Omega_{2}$ is an univalent in U and satisfy the bellow inequality:

$$1+\theta \chi_{1}\left(z\right)+\frac{\omega z{\chi_{1}}^{\prime }\left(z\right)}{\chi_{1}\left(z\right)}≺\Omega_{1}\left(z\right)\;≺\;1+\theta \chi_{2}\left(z\right)+\frac{\omega z{\chi_{2}}^{\prime }\left(z\right)}{\chi_{2}\left(z\right)},$$

then

$$\chi_{1}\left(z\right)≺{\left(\frac{l_{1}T_{r,k}^{\lambda -\frac{k}{r},\delta ,\sigma }\left(f*\varphi \right)\left(z\right)+l_{2}T_{r,k}^{\lambda ,\delta ,\sigma }\left(f*\varrho \right)\left(z\right)\;}{\left(l_{1}+l_{2}\right)z^{p}}\right)}^{\gamma }≺\chi_{2}\left(z\right),$$

and the dominant $\chi_{2}$ (the subordinant $\chi_{1}$) is besting.

## Class of admissible functions involving $T_{z}^{\lambda ,\delta ,\sigma }$

Below, we provide a definition of a class of admissible functions that includes the operator $T_{z}^{\lambda ,\delta ,\sigma }$, we also present some results related to this definition.

Definition 5.1 Let Ω⊂C, χ∈Q, and $n\in Z^{+}$. The class of admissible functions $L_{p}\;\left[\Omega ,\chi \right]$ consists of those functions $\phi :C^{3}\times U\to C$ that satisfy the admissibility condition:

(5.1) ϕ(a,b,c;z)∉Ω

whenever

$$a=\chi \left(g\right),\;b=\frac{mg\chi^{\prime }\left(g\right)+\left(\lambda +\sigma \right)\chi \left(g\right)}{p+\lambda +\sigma },$$

and

$$Re\left(\frac{c\left(p+\lambda +\sigma -1\right)\left(p+\lambda +\sigma \right)-b\left(p+\lambda +\sigma \right)\left(2\lambda +2\sigma -1\right)-3a{\left(\lambda +\sigma \right)}^{2}}{b\left(p+\lambda +\sigma \right)-a\left(\lambda +\sigma \right)}\right)\geq mRe\left(\frac{g\chi^{\prime \prime }\left(g\right)}{\chi^{\prime }\left(g\right)}+1\right),$$

z∈U,g∈∂U∖E(χ),andm≥p.

Theorem 5.1 *Let*$f\in A_{p}$*and*$\chi \in Q_{0}\cap H\;\left[0,p\right]$*. If*$\phi \in L_{p}\;\left[\Omega ,q\right]$*and*

(5.2) $$\phi \left(T_{z}^{\lambda ,\delta ,\sigma }f\left(z\right),T_{z}^{\lambda -1,\delta ,\sigma }f\left(z\right),T_{z}^{\lambda -2,\delta ,\sigma }f\left(z\right);z\right)\in \Omega ,\;$$

then

$$T_{z}^{\lambda ,\delta ,\sigma }f\left(z\right)≺\chi \left(z\right).$$

**Proof.** Define

(5.3) $$k\left(z\right)=T_{z}^{\lambda ,\delta ,\sigma }f\left(z\right),\;\left(z\in U\right).$$

Differentiating 5.3 with respect to z and use (2.11), we obtain

(5.4) $$T_{z}^{\lambda -1,\delta ,\sigma }f\left(z\right)=\frac{zk^{\prime }\left(z\right)+\left(\lambda +\sigma \right)k\left(z\right)}{p+\lambda +\sigma }.\;$$

By using the same argument to calculation of 5.4, we have

(5.5) $$T_{z}^{\lambda -2,\delta ,\sigma }f\left(z\right)=\frac{z^{2}k^{\prime \prime }\left(z\right)+2\left(\lambda +\sigma \right)zk^{\prime }\left(z\right)+\left(\lambda +\sigma -1\right)\left(\lambda +\sigma \right)k\left(z\right)}{\left(p+\lambda +\sigma -1\right)\left(p+\lambda +\sigma \right)}.\;$$

Define the following transformations from $C^{3}$ into C

$$a\left(r,s,t\right)=r,\;b\left(r,s,t\right)=\frac{s+\left(\lambda +\sigma \right)r}{p+\lambda +\sigma },\;\text{and}$$

(5.6) $$c\left(r,s,t\right)=\frac{t+2\left(\lambda +\sigma \right)s+\left(\lambda +\sigma -1\right)\left(\lambda +\sigma \right)r}{\left(p+\lambda +\sigma -1\right)\left(p+\lambda +\sigma \right)}\;.\;$$

Let $\psi :C^{3}\times U\to C$ is given by

ψ(a,b,c;z)=ϕ(a,b,c;z)

(5.7) $$=\phi \left(r,\frac{s+\left(\lambda +\sigma \right)r}{p+\lambda +\sigma },\frac{t+2\left(\lambda +\sigma \right)s+\left(\lambda +\sigma -1\right)\left(\lambda +\sigma \right)r}{\left(p+\lambda +\sigma -1\right)\left(p+\lambda +\sigma \right)};z\right).$$

In view of 5.3–5.6, the Eq. (5.7) leads to

$$\psi \left(k\left(z\right),zk^{\prime }\left(z\right),z^{2}k^{\prime \prime }\left(z\right);z\right)=\phi \left(T_{z}^{\lambda ,\delta ,\sigma }f\left(z\right),T_{z}^{\lambda -1,\delta ,\sigma }f\left(z\right),T_{z}^{\lambda -2,\delta ,\sigma }f\left(z\right);z\right),$$

so it follows from 5.2 that

$$\psi \left(k\left(z\right),zk^{\prime }\left(z\right),z^{2}k^{\prime \prime }\left(z\right);z\right)\in \Omega .$$

Moreover,

$$Re\left(\frac{t}{s}+1\right)=Re\left(\frac{c\left(p+\lambda +\sigma -1\right)\left(p+\lambda +\sigma \right)-b\left(p+\lambda +\sigma \right)\left(2\lambda +2\sigma -1\right)-3a{\left(\lambda +\sigma \right)}^{2}}{b\left(p+\lambda +\sigma \right)-a\left(\lambda +\sigma \right)}\right)$$

$$\;\geq mRe\left(\frac{g\chi^{\prime \prime }\left(g\right)}{\chi^{\prime }\left(g\right)}+1\right).$$

Consequently, the classes $\Psi_{p}\;\left[\Omega ,\chi \right]$ and $L_{p}\;\left[\Omega ,\chi \right]$ are equivalent (from Definition 1.2 and Definition 5.1). According to 5.2, from Theorem 1.1, we obtain

$$T_{z}^{\lambda ,\delta ,\sigma }f\left(z\right)≺\chi \left(z\right).$$

Therefore, our theorem is proven.

Taking $\chi \left(z\right)=\frac{1+z}{1-z}$ in the Definition 5.1, the conditions of the class $L_{p}\;\left[\Omega ,\chi \right]$ become

$$\phi \left(\rho i,\frac{\tau +\left(\lambda +\sigma \right)\rho i}{p+\lambda +\sigma },\frac{\left(\mu +i\nu \right)+2\left(\lambda +\sigma \right)\tau +\left(\lambda +\sigma -1\right)\left(\lambda +\sigma \right)\rho i}{\left(p+\lambda +\sigma -1\right)\left(p+\lambda +\sigma \right)};z\right)\notin \Omega ,$$

where ρ,τ,μ,ν∈R, τ+μ≤0, $\tau \leq -\frac{p}{2}\left(1+\rho^{2}\right)$, and z∈U. In this particular case Theorem 5.1 become

Corollary 5.1 *Let*$f\in A_{p}$*. If*$\phi \in L_{p}\;\left[\Omega ,q\right]$*and*

$$\phi \left(T_{z}^{\lambda ,\delta ,\sigma }f\left(z\right),T_{z}^{\lambda -1,\delta ,\sigma }f\left(z\right),T_{z}^{\lambda -2,\delta ,\sigma }f\left(z\right);z\right)\in \Omega ,$$

then

$$Re\left(T_{z}^{\lambda ,\delta ,\sigma }f\left(z\right)\right)>0.$$

Theorem 5.2 *Let*$f\in A_{p}$*. Then*

$$Re\left(\frac{T_{z}^{\lambda ,\delta ,\sigma }f\left(z\right)+p}{2}+\frac{z{\left(T_{z}^{\lambda ,\delta ,\sigma }f\left(z\right)\right)}^{\prime }}{T_{z}^{\lambda ,\delta ,\sigma }f\left(z\right)+1}\right)>0,$$

implies that

$$Re\left(T_{z}^{\lambda ,\delta ,\sigma }f\left(z\right)\right)>0.$$

**Proof.** It follows from (2.11) that

$$\frac{T_{z}^{\lambda ,\delta ,\sigma }f\left(z\right)+p}{2}+\frac{z{\left(T_{z}^{\lambda ,\delta ,\sigma }f\left(z\right)\right)}^{\prime }}{T_{z}^{\lambda ,\delta ,\sigma }f\left(z\right)+1}=\frac{T_{z}^{\lambda ,\delta ,\sigma }f\left(z\right)+p}{2}+\frac{\left(p+\lambda +\sigma \right)T_{z}^{\lambda -1,\delta ,\sigma }f\left(z\right)-\left(\lambda +\sigma \right)T_{z}^{\lambda ,\delta ,\sigma }f\left(z\right)}{T_{z}^{\lambda ,\delta ,\sigma }f\left(z\right)+1}.$$

Put Ω={w:Re(w)>0}, and

$$\phi \left(T_{z}^{\lambda ,\delta ,\sigma }f\left(z\right),\;T_{z}^{\lambda ,\delta ,\sigma }f\left(z\right);z\right)=\frac{T_{z}^{\lambda ,\delta ,\sigma }f\left(z\right)+p}{2}+\frac{\left(p+\lambda +\sigma \right)T_{z}^{\lambda -1,\delta ,\sigma }f\left(z\right)-\left(\lambda +\sigma \right)T_{z}^{\lambda ,\delta ,\sigma }f\left(z\right)}{T_{z}^{\lambda ,\delta ,\sigma }f\left(z\right)+1}.$$

Hence,

$$\phi \left(T_{z}^{\lambda ,\delta ,\sigma }f\left(z\right),\;T_{z}^{\lambda ,\delta ,\sigma }f\left(z\right);z\right)\in \Omega .$$

Therefore, we need only show that $\phi \in L_{p}\;\left[\Omega ,q\right]$ and apply Corollary 5.1. Indeed, this is sufficient to show that

$$\phi \left(\rho i,\frac{\tau +\left(\lambda +\sigma \right)\rho i}{p+\lambda +\sigma };z\right)\notin \Omega .$$

This follows immediately since

$$Re\left(\phi \left(\rho i,\frac{\tau +\left(\lambda +\sigma \right)\rho i}{p+\lambda +\sigma };z\right)\right)=Re\left(\frac{\rho i+p}{2}+\frac{\left(p+\lambda +\sigma \right)\frac{\tau +\left(\lambda +\sigma \right)\rho i}{\left(p+\lambda +\sigma \right)}-\left(\lambda +\sigma \right)\rho i}{\rho i+1}\right)$$

$$=\frac{p}{2}+\frac{\tau }{1+\rho^{2}}\leq 0$$

as required.

## Applications to fluid mechanics

The phenomenon of vortex motion in fluid dynamics occurs when gases or liquids rotate around a specific center, and it is considered significant in numerous applications. It has a notable impact on atmospheric systems and marine engineering. Vortices are present in various forms, from natural hurricanes to tarbines in engineering applications.

Zayed at al. studied vortex dynamics inside a cylindrical container with a radius of ρ in [6,13], where they clarified that the vortex can be represented as ξ(z) and the cylinder surface as χ(z), then it is required to show that ξ(z)≺χ(z), (z∈U). In vortex motion, the object and cloak correspond to a vortex within the circular cylinder and the cylinder's surface, respectively, with the latter rendering the object invisible to an observer. This paper examines the vortex within an infinite circular cylinder of radius ρ, with the flow described by the following equations:

(1) The velocity of a source of radius r with a flow rate of strength κ is expressed as $\nu_{\rho }=0.$
(2) The tangential velocity is zero, specifically, $\nu_{\vartheta }=-\kappa /2\pi \rho$.

Considering the Cauchy-Riemann equations, we get

$$\nu_{\rho }=\frac{\partial A}{\partial \rho }=\frac{1}{\rho }\frac{\partial B}{\partial \vartheta },\;\nu_{\vartheta }=\frac{1}{\rho }\frac{\partial A}{\partial \vartheta }=-\frac{\partial B}{\partial \rho }.$$

Consequently, the potential, stream, and complex potential functions for the source are as follows:

$$A=-\frac{\kappa }{2\pi }\vartheta \;\text{and}\;B=\frac{\kappa }{2\pi }\log r,$$

Therefore,

$$T=A+\text{iB}=\frac{i\kappa }{2\pi \rho }\log z.$$

It is obvious that

$$\frac{\text{dT}}{\text{dz}}=\frac{i\kappa }{2\pi \left(z-z\left(0\right)\right)}\;\left(\;z\left(0\right)\;\text{is}\;a\;\text{situated}\;\text{source}\right).$$

Both the vortices within the circular cylinder and the surface of the cylinder are simply connected regions in the complex plane; thus, both regions are identical to conformal mappings on the open unit disk U.In our work, we have presented several results confirming ξ(z)≺χ(z), (z∈U) as in Theorem 3.1, 3.2, 3.3, 3.4, and 5.1.

**Conclusion.** In summary, we have effectively extended Srivastava's fractional integral calculus by using the (r,k)-gamma function and the (r,k)-hypergeometric function. Fundamental results pertaining to the (r,k)-hypergeometric function were established, providing new insights and expanding the existing structure of fractional calculus. Furthermore, the sandwich results for p-valent analytic functions have been presented in the context of subordination and superordination. The process of generalization involved the empoly of the (r,k)-gamma function to reformulate the fractional integral operator, including it into the structure of the (r,k)-hypergeometric function. The scope of fractional calculus and its applications have been broadened in complex analysis by this novel approach. The results derived from the theories of subordination and superordination further demonstrate the efficacy of the new fractional operator in formulating sandwich theorems. Finally, our work paves the way for subsequent studies to explore additional characteristics and applications of the (r,k)*-*hypergeometric function, thereby contributing to the advancement of mathematical analysis in fractional calculus and its related fields. Future research will concentrate on investigating new classes of analytic functions that incorporate conditions pertaining to our fractional integral operator. Consequently, these will be closely linked to the results articulated in our work. The research scope will be broadened to encompass results related to partial derivatives in the context of multi-variable complex functions. We also urge scholars to utilize our fractional integral operator in the examination of specific subclasses of bi-univalent functions. Additionally, we advocate for the definition and derivation of novel conclusions pertaining to the fractional differential operator, which is dual to $T_{z}^{\lambda ,\delta ,\sigma }$.

## Method validation

Not applicable.

## Limitations

None.

## Ethics statements

Not applicable.

## CRediT author statement

**Adel Salim Tayyah:** contributed to the design and implementation of the research, conducted the experiments, and drafted the initial manuscript. Meanwhile, **Waggas Galib Atshan:** provided supervision for the research project, offered critical guidance throughout the study, and assisted in reviewing the final manuscript.

## Declaration of competing interest

The authors declare that they have no known competing financial interests or personal relationships that could have appeared to influence the work reported in this paper.

## Data Availability

No data was used for the research described in the article.

## References

[1] Miller S.S., Mocanu P.T., Reade M.O. (1974). Bazilevic functions and generalized convexity. Rev. Rumaine Math. Pures Appl..

[2] Seoudy T.M. (2023). Some applications of third-order differential subordination for analytic functions involving k-Ruscheweyh derivative operator. Afrika Mat.

[3] Sharma M., Jain N.K., Kumar S. (2024). Starlikeness of analytic functions using special functions and subordination. Boletín la Soc. Matemática Mex..

[4] Saliu A., Jabeen K., Ravichandran V. (2024). Differential subordination for certain strongly starlike functions. Rend. del Circ. Mat. di Palermo Ser. 2.

[5] Ali E.E., Srivastava H.M., El-Ashwah R.M., Albalahi A.M. (2022). Differential subordination and differential superordination for classes of admissible multivalent functions associated with a linear operator. Mathematics.

[6] Morais J., Zayed H.M. (2021). Applications of differential subordination and superordination theorems to fluid mechanics involving a fractional higher-order integral operator. Alexandria Eng. J..

[7] Tang H., Srivastava H.M., Deng G.-T., Li S.-H. (2017). Second-order differential superordination for analytic functions in the upper half-plane. J. Nonlinear Sci. Appl..

[8] El-Deeb S.M., Bulboacă T. (2021). Differential Sandwich-Type Results for Symmetric Functions Associated with Pascal Distribution Series. J. Contemp. Math. Anal. (Armenian Acad. Sci.).

[9] Aouf M.K., Mostafa A.O. (2020). Subordination results for analytic functions associated with fractional q-calculus operators with complex order. Afrika Mat.

[10] Seoudy T.M. (2021). Second order differential subordination and superordination of Liu-Srivastava operator on meromorphic functions. Afrika Mat.

[11] Aouf M.K., Madian S.M. (2021). Subordination factor sequence results for starlike and convex classes defined by q-Cătaş operator. Afrika Mat.

[12] Shakir Q.A., Tayyah A.S., Breaz D., Cotîrlă L.-I., Rapeanu E., Sakar F.M. (2024). Upper Bounds of the Third Hankel Determinant for Bi-Univalent Functions in Crescent-Shaped Domains. Symmetry (Basel).

[13] Al-Refai M. (2020). On weighted Atangana–Baleanu fractional operators. Adv. Differ. Equations.

[14] Aldawish I., Ibrahim R.W. (2023). Studies on a new K-symbol analytic functions generated by a modified K-symbol Riemann-Liouville fractional calculus. MethodsX.

[15] Ragoub L., Gómez-Aguilar J.F., Pérez-Careta E., Baleanu D. (2024). On a class of Lyapunov's inequality involving λ-Hilfer Hadamard fractional derivative. AIMS Mathematics.

[16] Poovarasan R., Gómez-Aguilar J.F., Govindaraj V. (2024). Investigating the existence, uniqueness, and stability of solutions in boundary value problem of fractional differential equations. Physica Scripta.

[17] Rahman G.U., Ahmad D., Gómez-Aguilar J.F., Agarwal R.P., Ali A. (2024). Study of Caputo fractional derivative and Riemann–Liouville integral with different orders and its application in multi-term differential equations. Math. Methods Appl. Sci..

[18] Zayed H.M., Mohammadein S.A., Aouf M.K. (2019). Sandwich results of p-valent functions defined by a generalized fractional derivative operator with application to vortex motion. Rev. la Real Acad. Ciencias Exactas, Físicas y Nat. Ser. A. Matemáticas.

[19] Amsheri S.M., Zharkova V. (2011). Differential sandwich theorems of ppp-valent functions associated with a certain fractional derivative operator. Kragujev. J. Math..

[20] Aouf M.K., Mostafa A.O., Zayed H.M. (2016). Subordination and superordination properties of p-valent functions defined by a generalized fractional differintegral operator. Quaest. Math..

[21] Aouf M.K., Mostafa A.O., Zayed H.M. (2016). Some Characterizations of integral operators associated with certain classes of p-valent functions defined by the Srivastava–Saigo–Owa fractional differintegral operator. Complex Anal. Oper. Theory.

[22] Tayyah A.S., Atshan W.G. (2024). New results on (r, k, μ)-Riemann–Liouville fractional operators in complex domain with applications. Fractal Fract.

[23] Srivastava H.M., Saigo M., Owa S. (1988). A class of distortion theorems involving certain operators of fractional calculus. J. Math. Anal. Appl..

[24] Aldawish I., Ibrahim R.W. (2023). Studies on a new K-symbol analytic functions generated by a modified K-symbol Riemann-Liouville fractional calculus. MethodsX.

[25] Yildiz Ç., Cotîrlă L.-I. (2023). Examining the Hermite–Hadamard inequalities for k-fractional operators using the Green function. Fractal Fract.

[26] Srivastava H.M., Aouf M.K. (1992). A certain fractional derivative operator and its applications to a new class of analytic and multivalent functions with negative coefficients. J. Math. Anal. Appl..

[27] Srivastava H.M., Aouf M.K. (1995). A certain fractional derivative operator and its applications to a new class of analytic and multivalent functions with negative coefficients, II. J. Math. Anal. Appl..

[28] Oros G.I., Oros G., Owa S. (2023). Subordination Properties of Certain Operators Concerning Fractional Integral and Libera Integral Operator. Fractal and Fractional.

[29] Andrews G.E., Askey R., Roy R., Roy R., Askey R. (1999).

[30] Miller S.S., Mocanu P.T. (2000).

[31] C. Pommerenke, Univalent Functions, Vandenhoeck and Rupercht, Germany, 1975.

[32] Bulboacă T. (2005). Differential subordinations and superordinations: recent results. Casa Cărt̨ii de S̨tiint̨ă.

[33] Miller S.S., Mocanu P.T. (2003). Subordinants of differential superordinations. Complex Var..

[34] Bulboacă T. (2002). Classes of first-order differential superordinations. Demonstr. Math..

[35] Bulboacǎ T. (2002). A class of superordination-preserving integral operators. Indag. Math..

[36] Ali R.M., Ravichandran V., Khan M.H., Subramanian K.G. (2004). Differential sandwich theorems for certain analytic functions. Far East J. Math. Sci..

[37] Miller S.S., Mocanu P.T. (1981). Differential subordinations and univalent functions. Michigan Math. J..

[38] Miller S.S., Mocanu P.T. (1987). Differential subordinations and inequalities in the complex plane. J. Differ. Equ..

[39] Srivastava H., Elashwah R., Kota W. (2018). Sandwich theorems for a class of p-valent meromorphic functions involving the Erdélyi-Kober-type integral operators. Turkish J. Math..

[40] El-Deeb S.M., Bulboacă T. (2019). Differential sandwich-type results for symmetric functions connected with a q-analog integral operator. Mathematics.

[41] Răducanu D., Nechita V.O. (2012). A differential sandwich theorem for analytic functions defined by the generalized Salagean operator. Aust. J. Math. Anal. Appl..

[42] Shammugam T.N., Ramachandran C., Darus M., Sivasubramanian S. (2007). Differential sandwich theorems for some subclasses of analytic functions involving a linear operator. Acta Math. Univ. Comenianae. New Ser..

[43] V. Ravichandran S.T.N., Sivasubramanian S. (2006). Differential sandwich theorems for some subclasses of analytic functions. Aust. J. Math. Anal. Appl..

[44] Shanmugam T.N., Sivasubramanian S., Silverman H. (2006). On sandwich theorems for some classes of analytic functions. Int. J. Math. Math. Sci..

[45] Atshan W.G., Battor A.H., Abaas A.F. (2021). Some sandwich theorems for meromorphic univalent functions defined by new integral operator. J. Interdiscip. Math..

[46] M. Saigo, A remark on integral operators involving the Gauss hypergeometric functions, 1978.

[47] J. Liouville, Mémoire sur le changement de la variable indépendante, dans le calcul des différentielles à indices quelconques, 1835.

[48] Ali H.M. (2019). New approximate solutions to fractional smoking model using the generalized Mittag-Leffler function method. Progr. Fract. Differ. Appl..

[49] T. Karite, A. Boutoulout, D.F.M. Torres, Enlarged controllability and optimal control of sub-diffusion processes with Caputo fractional derivatives, arXiv Prepr. arXiv1911.10199, 2019.

[50] Srivastava H.M., Bansal M.K., Harjule P. (2024). A class of fractional integral operators involving a certain general multiindex Mittag-Leffler function. Ukr. Math. J..

[51] Marx A. (1933). Untersuchungen über schlichte Abbildungen. Math. Ann..

[52] Strohhäcker E. (1933). Beiträge zur Theorie der schlichten Funktionen. Math. Zeitschrift.

